# Precision Oncology in Sarcomas: Divide and Conquer

**DOI:** 10.1200/PO.18.00247

**Published:** 2019-04-25

**Authors:** Roberto Carmagnani Pestana, Roman Groisberg, Jason Roszik, Vivek Subbiah

**Affiliations:** ^1^ The University of Texas MD Anderson Cancer Center, Houston, TX

## Abstract

Sarcomas are a heterogeneous group of rare malignancies that exhibit remarkable heterogeneity, with more than 50 subtypes recognized. Advances in next-generation sequencing technology have resulted in the discovery of genetic events in these mesenchymal tumors, which in addition to enhancing understanding of the biology, have opened up avenues for molecularly targeted therapy and immunotherapy. This review focuses on how incorporation of next-generation sequencing has affected drug development in sarcomas and strategies for optimizing precision oncology for these rare cancers. In a significant percentage of soft tissue sarcomas, which represent up to 40% of all sarcomas, specific driver molecular abnormalities have been identified. The challenge to evaluate these mutations across rare cancer subtypes requires the careful characterization of these genetic alterations to further define compelling drivers with therapeutic implications. Novel models of clinical trial design also are needed. This shift would entail sustained efforts by the sarcoma community to move from one-size-fits-all trials, in which all sarcomas are treated similarly, to divide-and-conquer subtype-specific strategies.

## INTRODUCTION

Sarcomas are a heterogeneous group of mesenchymal malignancies that comprise less than 1% of adult and 12% of pediatric cancers.^1,2^ The WHO has defined more than 50 sarcoma subtypes, including a wide array of tumors that arise from adipose, muscular, bone, cartilage, and vascular tissues.^3^ Treatment options for patients with advanced soft tissue sarcomas (STSs) are limited, and a one-size-fits-all paradigm has prevailed despite the diverse nature of STSs. For metastatic STSs, anthracycline-based chemotherapy remains the backbone of first-line treatment regimens.^4^ However, efficacy is limited, with a median progression-free survival (mPFS) of approximately 6 months, and the incidence of treatment-associated adverse events (AEs) is high.^5^ Therefore, more individualized treatment options are critical to improve the survival and quality of life of this patient population.

Reflecting STS heterogeneity, multiple molecular pathways are implicated in the development and progression of these cancers. The characterization of specific genetic aberrations has led to the identification of novel diagnostic, prognostic, and predictive biomarkers.^6^ An understanding of the prevalence and function of specific genetic alterations in STS subtypes is critical to developing more-effective diagnostic tests and therapeutic approaches. Targeted therapies have the potential to produce significant tumor response by disrupting molecular pathways that drive oncogenesis, thus providing highly personalized treatment.^7^ A recent report by Lucchesi et al^8^ analyzed 584 patients with STS in the American Association for Cancer Research (AACR) Project Genomics Evidence Neoplasia Information Exchange (GENIE) database and identified that 41% of patients harbored a genetic alteration with potential to influence therapy. Accordingly, a single-center report by Boddu et al^9^ analyzed 114 patients with sarcoma and identified that 49% carried a mutation deemed actionable; 15 patients had next-generation sequencing (NGS)–guided therapies, with 26% of these achieving clinical benefit (Table 1). This review focuses on how incorporation of NGS has affected drug development in sarcomas and strategies for optimizing precision oncology for these rare cancers.

**TABLE 1.**
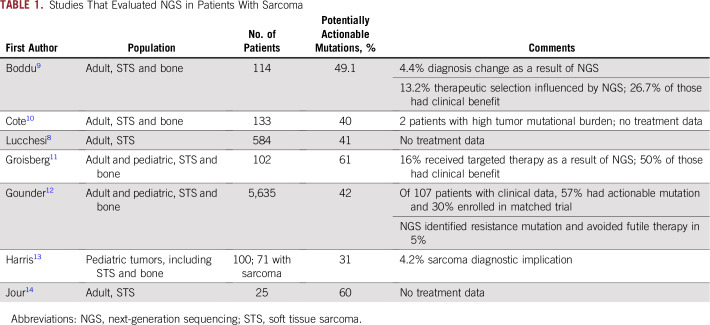
Studies That Evaluated NGS in Patients With Sarcoma

## SARCOMA SUBTYPES

**FIG 1.**
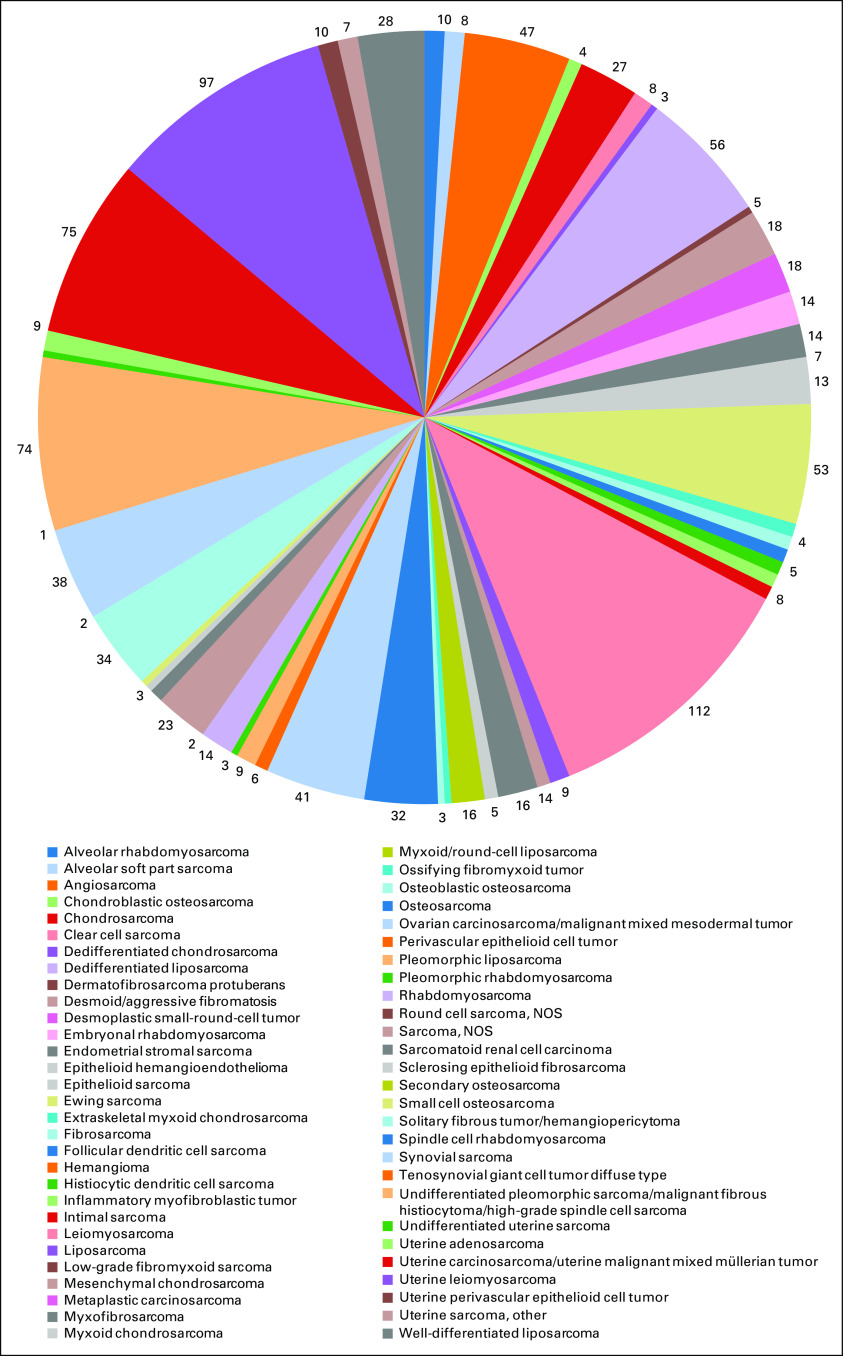
Prevalent sarcoma subtypes in the Association for Cancer Research Project Genomics Evidence Neoplasia Information Exchange database (n = 584). NOS, not otherwise specified.

Traditionally, STSs have been classified on the basis of a presumptive tissue of origin or unique architectural pattern.^3^ However, molecular diagnostics have enhanced our understanding of the complex morphologic-genetic associations of STSs, establishing the foundation for a precision approach to therapy. Indeed, the current classification system was designed with meticulous correlation of recurrent molecular alterations with discrete histologic subtypes.^15^ Of the STS subtypes, the most prevalent in adults are undifferentiated/unclassified STS, undifferentiated pleomorphic sarcoma (UPS), liposarcoma, and leiomyosarcoma (Fig 1).

A number of subtypes are associated with specific chromosomal translocations. Molecular approaches to detect the protein products of these fusion genes, such as fluorescence in situ hybridization and reverse transcriptase polymerase chain reaction, can aid in the diagnosis of these tumors. For example, myxoid liposarcomas are characterized by reciprocal t(12;16)(q32;q16) translocation between the *DDIT3*(*CHOP)* and *FUS* genes, and synovial sarcomas demonstrate the translocation t(X;18)(p11.2;q11.2), which results in the fusion of the *SS18* and *SSX* genes.^16^ A prospective, multicenter, French Sarcoma Group observational study demonstrated that diagnosis was modified after molecular genetics analysis for 53 (14%) of 384 patients with STS, and it has been argued that such testing should be considered mandatory.^17^

Previous studies have described the occurrence of potential driver mutations in a significant fraction of patients with sarcoma (Table 2), and these alterations are often type specific.^8,18^ For illustration, well-differentiated/dedifferentiated liposarcomas have a simple genomic profile characterized by amplification of the *MDM2* and *CDK4* genes.^19^ Alternatively, *ALK* fusions are observed in 50% of inflammatory myofibroblastic tumor (IMTs), whereas a subset of non-ALK–rearranged IMTs carry fusions in other tyrosine kinases, such as ROS1 or NTRK.^20,21^ These examples emphasize the heterogeneity of STSs and the importance of understanding the underlying biologic differences to enable rational therapeutic interventions.

**TABLE 2.**
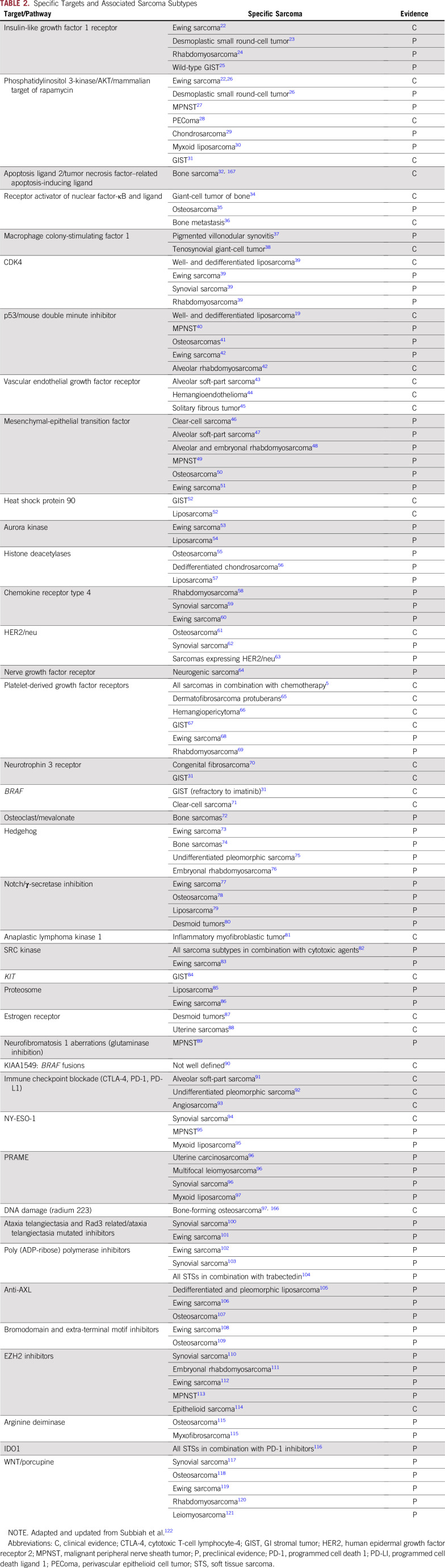
Specific Targets and Associated Sarcoma Subtypes

## CONVENTIONAL THERAPY

For most patients with advanced STS, systemic therapy is administered with palliative intent to alleviate symptoms and extend overall survival (OS). The majority of clinical trials that have assessed chemotherapy for advanced STSs have included heterogeneous populations, which complicates the assessment of clinical activity in specific subtypes.

Anthracyclines remain the most-used agents in the first-line treatment of metastatic STS. Several phase II/III trials have evaluated the efficacy of doxorubicin as a single agent, with objective response rates (ORRs) of approximately 10% to 30% and median OS of 8 to 17 months.^123-125^ In most prospective trials, anthracycline-based combination regimens, such as doxorubicin with ifosfamide, are associated with a higher ORR compared with single-agent doxorubicin, although in general, combinations have not prolonged OS.^125^ One exception is the combination of doxorubicin and olaratumab, which led to improved OS compared with doxorubicin as a single agent in a randomized phase II trial (26.5 *v* 14.7 months).^5^ Of note, these anthracycline-containing regimens are associated with considerable toxicity, with up to 70% of patients experiencing grade-3-or-higher AEs.^125^ The combination of gemcitabine and docetaxel is another widely used regimen for the treatment of STS.^124,126^ In a retrospective analysis by the French Sarcoma Group that involved 133 patients with STSs (leiomyosarcomas, [n = 76]; other subtypes, [n = 57]) treated with gemcitabine and docetaxel, the ORR was 18% and median OS was 12 months.^127^

Eribulin, trabectedin, and pazopanib are approved as later lines of treatment. Eribulin, an antimicrotubule agent, was shown to improve median OS in a multicenter phase III trial with dacarbazine as control.^128^ Of interest, benefit was limited to patients with liposarcoma.^128^ Trabectedin, a synthetic alkaloid derived from the Caribbean tunicate *Ecteinascidia turbinata*, was approved by the Food and Drug Administration (FDA) for patients with advanced liposarcoma and leiomyosarcoma who received prior anthracycline-based regimens on the basis of a phase II clinical trial that demonstrated its superiority to dacarbazine.^129^ Retrospective reports have shown particular benefit of therapy with trabectedin for myxoid liposarcoma (ORR, 51%; disease control rate [DCR], 90%), which achieved an mPFS of 14 months in patients who received multiple lines of treatment.^130^ Pazopanib is a multitargeted, small-molecule tyrosine kinase inhibitor (TKI). A randomized phase III trial compared pazopanib with placebo in patients with a variety of STS subtypes who had experienced disease progression after first-line anthracycline-containing therapy; a significantly prolonged mPFS in the pazopanib arm (4.6 *v* 1.6 months) was demonstrated, with benefit consistent across subgroups.^131,132^

## SARCOMA FRUIT BOWL THEORY: DIVIDE AND CONQUER

**FIG 2.**
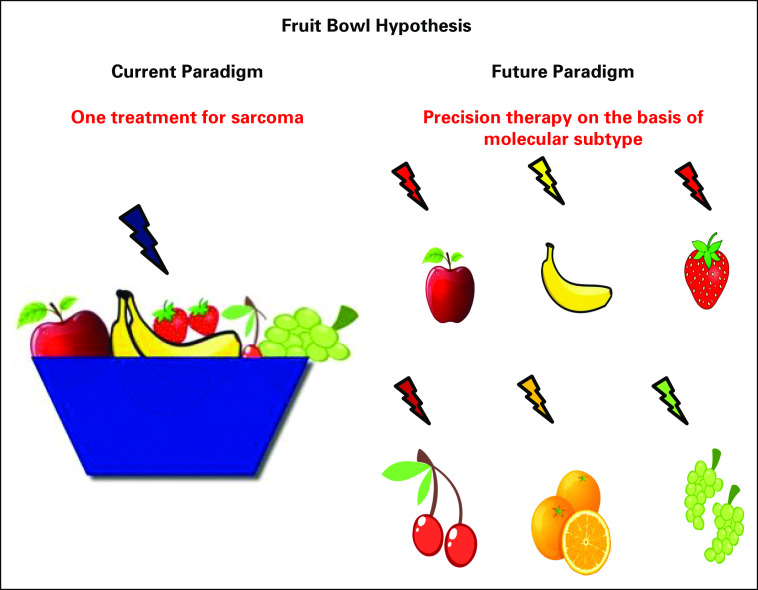
Fruit bowl theory for customized therapy in sarcoma.

Clinical trial design and, thus, treatment strategies need to expand as our capability of examining the genome, proteome, transcriptome, and immunophenotype of tumors continues to advance. With the increase in our understanding of the molecular underpinnings of sarcoma, the time is right to divide and conquer. This will allow us to move away from the one-size-fits-all or fruit bowl theory (the current paradigm) in which most patients with sarcoma receive conventional treatment that is not based on molecular testing to individual, customized therapy for specific sarcoma subtypes on the basis of biology (the future paradigm; Fig 2).^133^ The challenge that could be posed under this new model is performing studies in rare subsets of an already rare disease. However, as has been recently demonstrated in other rare tumor types that have led to FDA-approved therapies, it is possible to establish efficacy with low patient numbers. For instance, vemurafenib was approved recently in BRAF^V600^-positive Erdheim-Chester disease on the basis of results in fewer than 25 patients.^134,135^

## STRATEGIES TO BUILD EVIDENCE

The evaluation of targeted therapies can be challenging when the mutations are rare and present in different histologies. Therefore, innovative clinical trial designs are required to determine clinical benefit of new therapies in patients with rare STS subtypes.

Basket trials are an emerging model of trial design centered on the assumption that the existence of a specific molecular aberration or biomarker predicts the benefit of targeted therapies, regardless of cancer tissue of origin.^136^ The success of basket trials predominantly depends on the strength of the evidence that demonstrates tumor dependence on the targeted pathways and on reliable inhibition of the target by the drug.^136^

One example is the basket trial of vemurafenib for tumors that harbor a *BRAF*^V600^ mutation.^71^ Vemurafenib is an orally available TKI of *BRAF*, with higher selectivity for the *BRAF*^V600^ mutant form, that had been approved previously for patients with *BRAF*^V600E^ mutation–positive metastatic melanoma.^137^ In this trial with six prespecified cohorts, there was one anecdotal response in a patient with clear-cell sarcoma. The ongoing National Cancer Institute Molecular Analysis for Therapy Choice trial is another ambitious illustration of novel trial strategies for the development of precision oncology; patients will be paired with a targeted drug that has demonstrated activity against their specific driver pathway abnormality, regardless of tumor histology.^18^

## NGS IN SARCOMAS AND TARGETED THERAPY

Massively parallel NGS technology is rapidly being adopted by researchers around the world. In sarcomas, it is being used predominantly as a shotgun-screening tool to seek out novel, recurrent, and potentially actionable mutations. This technology is proving useful in the study of rare tumors because even a few patient samples can yield tremendous understanding of the disease on a molecular level. Some investigators are moving beyond searching for driver mutations and instead asking whether NGS can predict response or resistance to therapy; others are creating expression profiles that go beyond individual genes. NGS has the potential to become, in the coming years, an established platform of choice for researchers who are studying sarcomas.

To date, many investigators have attempted to identify recurrent aberrations using NGS. On the basis of the large variety of identified mutations in cancer-associated pathways, these are predominantly secondary mutations that occur later in the course of tumorigenesis. They may be responsible for accelerated growth in advanced disease but are unlikely to be the sole cause of the malignancy. Identification of these mutations is important, and with so much diversity in involved pathways, an individualized approach is necessary for proper sarcoma treatment.

The majority of mutations detected by NGS will not be drivers and, as such, will not translate into clinical benefit for patients. However, as a result of the decreasing costs of NGS-based commercially available genomic profiling, clinicians are identifying driver mutations and exploiting their therapeutic benefit. For patients with few treatment options, a rationally chosen clinical trial on the basis of NGS-derived data is the best chance for prolonged survival.

To demonstrate the ability of NGS to detect gene fusions, Qadir et al^138^ designed Child-Seq, a pilot platform that rapidly screens cancer tissue for particular gene fusions characteristic of specific sarcomas. Results demonstrated that the technique could reliably identify gene fusions in their corresponding tumor type without false positives. Although this platform was limited to four particular childhood sarcomas, it is legitimate to conceptualize a more robust tool that could identify dozens or even hundreds of fusions. In fact, currently there are commercially available tools for molecular tissue testing that include detection of fusions; identification of pathognomonic genetic alterations could enable a clinical diagnosis that is based solely on sequencing data. The potential to identify fusions by cell-free DNA testing also is promising. Shukla et al^139^ demonstrated the feasibility of detecting *EWSR1* fusions in plasma derived cell-free DNA from patients with Ewing sarcoma and desmoplastic small round-cell tumor.

In general, NGS has proven to be a highly reliable technology. However, Varga et al^140^ reported a case of undifferentiated sarcoma in which a 236-gene NGS panel showed pathogenic mutations in *BRCA2* and *MLH1*. The patient was counseled about the implications for herself and her family members. However, Sanger sequencing of the patient’s tumor as well as her father’s germline testing revealed that the *MLH1* mutation was a variant of unknown significance rather than a pathogenic mutation. Additional discussions with the NGS laboratory revealed that its algorithms had flagged the mutation for review, but human error led to the release of this erroneous finding. Although this is a single case report, it highlights that even very robust technologies are subject to error.

### NGS for Prediction

NGS has the potential to identify specific molecular subtypes with particular sensitivity or resistance to approved treatments. To date, however, there have been few examples of NGS being used as a predictive tool in sarcomas, and it important to recognize that the examples discussed herein are preliminary and should be interpreted with caution. Undoubtedly, as more patients undergo sequencing as part of their clinical care, patterns of response to therapy will emerge.

Koehler et al^141^ retrospectively reviewed 19 patients treated with pazopanib who also received NGS of their tumors. The authors found that the mPFS was significantly longer in patients with *TP53* mutant advanced STS (208 days) than in those with *TP53* wild-type tumors (136 days).

Lim et al^142^ performed NGS-based comprehensive genomic profiling on 39 paired samples from tumor and normal tissue from patients treated with everolimus. The patients’ tumor histology varied, but seven patients with sarcomas were included. The authors reported 22 patients with clinical benefit after treatment with everolimus, and 10 of these patients had aberrations in the mammalian target of rapamycin signaling pathway. Conversely, patients with mutations in chromatin remodeling genes and in receptor tyrosine kinase signaling universally did not respond to everolimus. These hypothesis-generating data reinforce the possibility of using NGS as a predictive tool in response to therapy.

### Designing Clinical Trials on the Basis of NGS

Wang et al^143^ performed a retrospective study of 75 patients with sarcomas who were referred to the Clinical Center for Targeted Therapy at MD Anderson Cancer Center. Patients underwent commercially available NGS of their tumors. Only 54 patients enrolled in a trial, but 13 were treated in multiple trials, with 93 total treatments given. Although few responses were seen, patients who received a gene aberration targeted therapy had a better DCR than those who received other types of therapy, which translated into significantly longer mPFS (5.8 *v* 1.9 months) and OS (15.9 *v* 8.7 months).

Chang et al^144^ performed complete multiomics studies on 59 childhood tumors referred to the National Institutes of Health. The majority of these tumors were sarcomas. Multiomics studies included whole-exome sequencing; germline, whole-transcriptome sequencing; and single-nucleotide polymorphism array analysis of the tumor. Germline cancer-associated gene mutations were reported in seven patients. The authors found targeted therapy–matched clinical trials for 44% of the patients on the basis of their mutational status. One patient had an epithelioid IMT that was identified to contain an RANBP2-anaplastic lymphoma kinase (ALK) fusion; he experienced a complete response to crizotinib for 8 months.

Harris et al^13^ applied the same approach as Chang et al^144^ to 89 pediatric tumors. As expected in a pediatric population, sarcomas were the most common malignancy. Patients underwent targeted NGS, first of 41 and then of 275 cancer-related genes. Treatment recommendations were made by multidisciplinary expert panel. The panel was able to make a targeted therapy recommendation in 31% of patients. Only three patients received matched targeted therapy, but none had an objective response. This lack of response may have been due to the lower-quality preclinical evidence on which two of the recommendations were based.

Worst et al^145^ conducted the first true prospective NGS-based trial with therapeutic intent. The Individualized Therapy for Relapsed Malignancies in Childhood study was a multicenter German effort to identify therapeutic targets in individual pediatric cancers. As with Harris et al,^13^ a multidisciplinary expert panel wrote a final recommended target report. In this pilot study, 57 patients were enrolled from 20 centers; approximately one half had sarcomas. One half of the patients harbored a potentially druggable alteration, and 10 patients went on to receive targeted therapy. The estimated final cost was relatively modest at approximately 7,000 euros. However, because this was a pilot study designed to assess feasibility, there was no follow-up of patients who were treated with personalized targeted therapy and outcomes were not reported. The authors described one example of an IMT that was found to have a CARS-ALK fusion. The patient was treated with an ALK inhibitor with at least a partial response and continued with treatment 26 months later. We hope that future iterations of this trial will follow patients longitudinally and report on PFS and OS.

## PRECISION IMMUNOTHERAPY FOR SARCOMAS

After more than a century since Coley’s report of complete regression of sarcomas secondary to severe episodes of erysipelas^146^, significant efforts have been made to incorporate immunotherapy in sarcoma treatment. Early results with the use of immune checkpoint inhibitors, however, have not been encouraging across all subtypes. Therefore, the development of immunotherapy for sarcomas also benefits from a precision oncology approach both in identifying predictive biomarkers and in developing strategies targeted to specific antigens.

In the phase II SARC028 trial (ClinicalTrials.gov identifier: NCT02301039) of pembrolizumab, an ORR of 18% was seen in STSs, with a 12-week PFS of 55%.^92,147^ Of note, this trial enrolled 40 patients with STS, including four tumor types; subgroup analysis of the results identified encouraging activity in UPS and dedifferentiated liposarcoma but not in other cohorts. The relevance of histology for immunotherapy efficacy in sarcomas has been highlighted by other reports. Combination of nivolumab and ipilimumab has provided promising efficacy in certain STS subtypes, with ORR and mPFS in the range of those produced by standard-of-care options; responses were observed in patients with leiomyosarcoma, myxofibrosarcoma, UPS, and angiosarcoma.^148^ Furthermore, activity in specific subtypes has been suggested by retrospective studies. In a case series, two patients with chordoma experienced responses to single-agent anti–programmed cell death ligand 1 (PD-L1) antibodies,^149^ and a review of patients enrolled in early-phase trials demonstrated that alveolar soft-part sarcoma was the most responsive subgroup to checkpoint blockade.^91,150^ The heterogeneity in the benefit of checkpoint inhibitors across sarcoma subtypes observed in these early-phase trials highlights the need for a precision approach to immunotherapy in these diverse tumors, and research to identify predictive biomarkers is warranted. One challenge is that at this time, the immune microenvironment of specific subtypes is not sufficiently characterized, and detailed characterization of the immune microenvironment in each subtype is a major task.

Examples of potential predictive biomarkers to allow for selection of sarcomas sensitive to immune checkpoint inhibition include microsatellite instability (MSI) status and PD-L1 expression in the tumor and microenvironment. In fact, one patient with sarcoma was included within the five trials that led to FDA approval of pembrolizumab for MSI-high tumors.^151^ A total of 149 patients were included in these trials, the majority (n = 90) with colorectal cancer, and results demonstrated an ORR of 39.6%, with 7.4% of patients achieving a complete response. In addition, responses were long lasting, with 78% lasting for 6 months or more.^151^ A recently published pan-cancer analysis identified that 5.7% of 785 STS cases analyzed were MSI high, which highlights the relevance of this indication for STS.^152^ With regard to PD-L1 status, a recent meta-analysis with data from 1,451 patients and 15 independent studies identified PD-L1 expression to be independently associated with poorer OS and event-free survival in bone and STSs.^153^ However, the predictive value of PD-L1 in selecting patients with sarcoma for immune checkpoint inhibition is still under evaluation, and clinical activity of checkpoint inhibitors in these trials were seen even in the absence of PD-L1 expression.^92^

Furthermore, cancer/testis antigens have been explored as potential targets for immunotherapy in STS and provide the utmost example of precision immunotherapy by targeting specific antigens. D’Angelo et al^94^ recently reported encouraging results with the adoptive transfer of NY-ESO-1 ^c259^T cells in 12 patients with synovial sarcoma. The ORR in this study was 50%, including one complete response, and mPFS was 15 weeks. Moreover, treatment was safe, and there were no fatal AEs. These results are promising and relevant for a larger population because NY-ESO-1 is expressed in 70% to 80% of synovial sarcomas and is present in myxoid liposarcoma and malignant peripheral nerve sheath tumors.^95,154^ Expanding on this concept, adoptive cell therapy targeting MAGE-A3, MAGE-A4, and MAGE-A10 also are under evaluation in clinical trials.

## GI STROMAL TUMOR: AN EXAMPLE OF THE PARADIGM SHIFT

Receptor tyrosine kinase activation in GI stromal tumor (GIST) is an emblematic example in which molecular characterization provides diagnostic, prognostic, and predictive information and enables improved outcomes.^155^ GISTs are intrinsically resistant to radiation and chemotherapy, being historically associated with poor prognosis because of the lack of effective treatment.^156^ For illustration, before the 1990s, recurrence rates reached 50% after resection, and median OS for metastatic GIST was approximately 9 months.^157^

**FIG 3.**
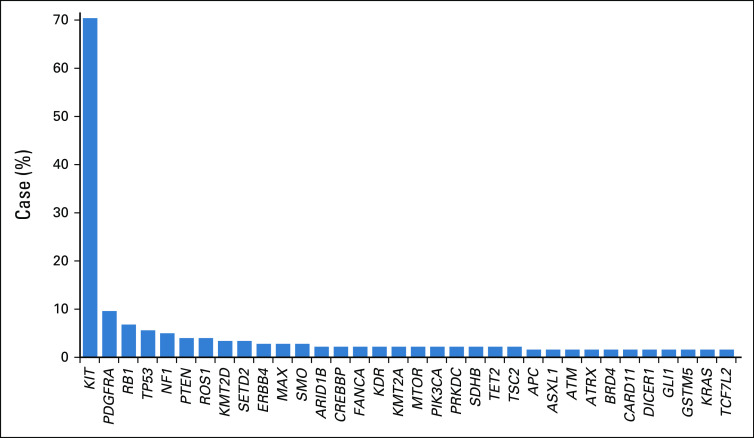
Mutation landscape of patients with GI stromal tumor in the Association for Cancer Research Project Genomics Evidence Neoplasia Information Exchange database (n = 169).

In the 1990s, the landmark description of gain-of-function mutations in *KIT* in five patients with GIST inaugurated a new era.^158^ Since then, many lines of evidence have also supported the causative relationship between *KIT* and GIST oncogenesis and have demonstrated that 70% to 85% of GISTs carry a *KIT* mutation that renders constitutionally activated kinase.^159^ The most common mutation occurs in exon 11, which encodes the juxtamembrane domain that physiologically inhibits the kinase activation loop.^159^ In addition, mutations have been described in exons 8 and 9, which encode the extracellular domains, and less frequently in exons 13 and 17, which encode the kinase domains.^159^ Approximately 30% of *KIT* wild-type GISTs harbor activating mutations in platelet-derived growth factor-α (PDGFRα).^67^ The majority of these mutations occur in exon 18, although mutations in exons 12 and 14 also have been described.^67^ Moreover, 10% to 15% of GISTs are wild type for *KIT*, and PDGFRα-BRAF mutations have been identified in 7% to 15% of these tumors, most commonly the exon 15 V600E mutation, and a small subset of these tumors are associated with loss of function of the succinate dehydrogenase respiratory chain complex.^31^ Figure 3 illustrates the mutation landscape of patients with GIST in the AACR Project GENIE database.

The development of molecular-targeted therapy has dramatically changed the prognosis for GIST; currently, median OS for metastatic disease is 5 years, and 26% to 35% of patients survive for 9 years.^160^ Overall, trials of imatinib have demonstrated a DCR of 70% to 85% for *KIT*-mutated GIST, with an mPFS of 20 to 24 months.^84^ TKIs have revolutionized the treatment of localized GIST as well. Adjuvant therapy with imatinib for 3 years is the current standard of care for patients at high risk of recurrence, which is based on phase III data that demonstrated improved 5-year recurrence-free survival and OS rates compared with patients who received imatinib for 1 year (recurrence-free survival, 65.6% *v* 47.9%; OS, 92.0% *v* 81.7%).^161^

Subanalyses of clinical trials also have been able to identify predictive factors of imatinib resistance. Patients with *KIT* exon 11 mutations have been identified as sensitive to imatinib, whereas patients with a *KIT* exon 9 mutation, identified in 10% to 20%, respond poorly to imatinib at standard doses.^155^

Most patients, however, eventually will develop resistance to treatment. In spite of the exceptional advances with first-line imatinib, the results with targeted treatments for later lines of therapy have been disappointing, emphasizing the need for new therapeutic approaches. Imatinib dose escalation is one option for patients who have experienced disease progression on imatinib, and approximately 20% remain progression free for 1 year at the higher dose, especially among those with exon 9 mutations.^162^ Furthermore, additional TKIs are under development to optimize the treatment of imatinib-resistant GIST. Demetri et al^163^ demonstrated that sunitinib, an orally available multitarget TKI, was active in patients with GIST who had disease progression on imatinib, achieving an mPFS of 27.3 weeks compared with 6.4 weeks for placebo. In addition, regorafenib was recently approved by the FDA as a third-line therapy and has shown promising activity in patients with exon 17 mutations, which confer resistance to imatinib and sunitinib. Avapritinib is currently under evaluation after having demonstrated significant activity in patients with PDGFRα D842V exon 18 mutation.^164,165^

**FIG 4.**
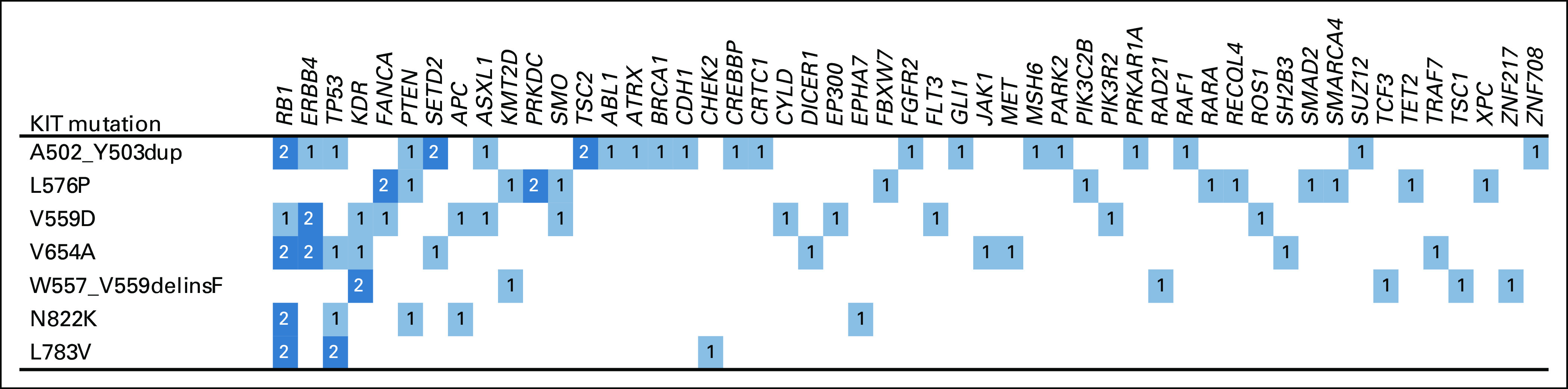
Co-occurring alterations in *KIT*-mutated GI stromal tumor in the Association for Cancer Research Project Genomics Evidence Neoplasia Information Exchange database (n = 31).

Available evidence indicates that *KIT* mutation is likely an early event in the development of GIST as suggested by the presence of activating *KIT* mutations in gastric precursor lesions. However, additional genetic mutations are needed for the progression of microscopic lesions to malignant GIST, and further characterization of these alterations may identify additional therapeutic targets. In a previous study, 74% of GISTs analyzed had at least one nondriver genetic abnormality.^164^ In that report, the most frequently mutated genes were *TP53*, *RB1*, *SETD2*, *PTEN*, *MAX*, *PIK3CA*, and *TSC1*, and the most prevalent copy number alteration in *KIT*-mutated GIST was *CDKN2A* deletion.^164^ In addition, activation of alternate receptor tyrosine kinases has been suggested as a mechanism of resistance to currently approved TKIs. For illustration, previous studies have identified that mutations that involve the *RB1* gene are associated with high-risk tumors and aggressive clinical behavior and that activating mutations in the RAS and phosphatidylinositol 3-kinase pathways contribute to TKI resistance.^166,167^ Figure 4 illustrates co-occurring genetic alterations in *KIT*-mutated GIST in the AACR Project GENIE database.

In conclusion, sarcomas are a rare group of mesenchymal tumors in which the integration of NGS for diagnosis and management has provided informative evidence for precision medicine. In a significant percentage of STSs, which represent up to 40% of all sarcomas, specific driver molecular abnormalities have been identified. The challenge to evaluate these mutations across rare cancer subtypes requires the careful characterization of these genetic alterations to further define compelling drivers with therapeutic implications as well as novel models of clinical trial design. This shift would entail sustained efforts by the sarcoma community to move from one-size-fits-all trials to divide-and-conquer subtype-specific strategies.
